# Relationship between irregular diet and risk of esophageal cancer: A meta-analysis

**DOI:** 10.3389/fgene.2022.1004665

**Published:** 2022-10-05

**Authors:** Jiayun Guan, Xixin Pan, Shenghang Ruan, Xiaopeng He, Yuhao Xu, Xiaoxiang Rong, Yanhua Ou

**Affiliations:** ^1^ The First School of Clinical Medicine, Southern Medical University, Guangzhou, China; ^2^ Department of Oncology, Nanfang Hospital, Southern Medical University, Guangzhou, China

**Keywords:** irregular diet, esophageal cancer, meta-analysis, systematic review, eating disorder

## Abstract

**Background:** Associations between irregular diet and the risk of esophageal cancer remain unclear. The current meta-analysis was performed to determine whether the presence of irregular diet increases the risk of esophageal cancer.

**Methods:** The data from PubMed, Cochrane Libraries, and Embase up to 23 January 2022 were included in our analysis to identify studies that investigated associations between irregular diet and the risk of esophageal cancer. Summary relative risk (RR) and 95% confidence intervals (CIs) were calculated using a random-effects model.

**Results:** Five cohort studies and one case-control study investigating associations between irregular diet and the risk of esophageal cancer were included. None of the articles demonstrated publication bias. The summary RR was 4.181 (95% CI 2.196–7.960, I^2^ = 66.1%, *p* = 0.011). In the subgroup analysis, we found significant heterogeneity in the Non-disease-causing group, nurse group and Asian group. The above three that produce heterogeneity may be the source of heterogeneity in the results of this study.

**Conclusion:** The current meta-analysis indicates that irregular diet increase the risk of esophageal cancer.

**Trial registration**: (https://www.crd.york.ac.uk/prospero/), (PROSPERO, CRD42022306407)

## Introduction

Esophageal cancer (EC) is the one of the most commonly diagnosed cancers and the sixth most common cause of cancer-related death worldwide (Sung et al., 2021). Genetic, environmental, and reproductive factors are currently believed to be associated with the pathogenesis of EC. Dietary factors such as irregular diet are also associated with EC incidence (Pan et al., 2019; Zhao et al., 2021). Irregular diet is when natural food intake patterns are disrupted and can include number of eating episodes, night-time fasting duration and time of first and last eating episodes (Srour et al., 2018; Xie et al., 2020). Meanwhile, irregular diet also referred to an unhealthy dietary behavior including overeating, unrestrained eating behavior, late night eating, etc (Kogevinas et al., 2018; Manoogian et al., 2019). On one hand, part of the irregular diet is not caused by disease, but because of stress, anxiety and tension, etc (Bogataj Jontez et al., 2021). On the other hand, the other part of irregular diet is caused by disease such as eating disorders (EDs), which is a serious psychological disorder, including anorexia nervosa (AN), bulimia nervosa (BN) and binge eating disorder (BED), etc (Nivedita et al., 2018; Yeh et al., 2021). Several studies reported that unrestrained eating behavior and not regular meal can increase the risk of esophageal cancer (Sun et al., 2010; Zhang et al., 2021).However, some articles have reported that certain irregular diet, such as eating dinner late, are not associated with the occurrence of esophageal cancer (Ibiebele et al., 2010). Therefore, whether irregular diet increases the incidence of esophageal cancer still lacks systematic cognition, thus it is very important to further explore associations between irregular diet and the risk of esophageal cancer.

It is controversial that whether irregular diet increase the risk of EC because of a lack of high-quality prospective studies. Thus, A meta-analysis investigating the relationship between irregular diet and EC is warranted is necessary. The vast majority of the existing systematic reviews are on the relationship between specific dietary patterns and esophageal cancer, or the relationship between dietary patterns and other cancers. However, to date no review or meta-analysis has investigated whether there is a relationship between irregular diet and EC risk. Furthermore, cohort studies and case-control studies investigating irregular diet and risk of esophageal cancer are rare. In the current cohort study, as well as in a case-control study, Lene Mellemkjaer et al. showed that anorexia increases the risk of esophageal cancer (Mellemkjaer et al., 2015), Yin Zhang et al. suggested unrestrained eating behavior was independently associated with increased risk of gastrointestinal tract cancers (Brewster et al., 2015), and David H. Brewster et al. thinked patients with eating disorders are at increased risk of developing EC (10). The aim of the current meta-analysis is to facilitate better clinical guidance by evaluating associations between irregular diet and EC.

## Methods

### Protocol and registration

This meta-analysis was conducted in compliance with the PRISMA statement (Page et al., 2021). The protocol was registered in PROSPERO (CRD42022306407). The full details of the protocol are available on request.

### Search strategy

765PubMed, Cochrane Libraries, and Embase databases were searched from inception to 23 January 2022. Key search terms included “anorexia nervosa” or “anorexia nervosas” or “eating” or “eating disorder” or “eating disorders” and “esophageal cancer” or “esophageal neoplasm” or “cancer of esophagus” or “cancer of the esophagus” or “esophagus cancer” or “esophagus cancers” or “esophageal neoplasms” or “esophageal cancers” or “cancers” or “cancer”. It is worth mentioning that we used controlled vocabulary (MeSH in Pubmed) and keywords as search terms and the full search strategy was added as supplementary materials (Supplementary Table S1). No language limitations were applied. We also manually searched references lists to prevent omissions.

### Inclusion criteria and exclusion criteria

The inclusion criteria included (Sung et al., 2021) studies that involved patients who had irregular diet (Zhao et al., 2021); research on associations between irregular diet and the risk of EC (Pan et al., 2019); hazard ratio (HR), odds ratio (OR), standardized incidence/hospitalization ratio (SIR/SHR), incidence rate ratio (IRR), and 95% confidence intervals (CIs) in the study were reported or could be calculated; and (Xie et al., 2020) case control studies or cohort studies.

The exclusion criteria included (Sung et al., 2021) reviews and comments (Zhao et al., 2021); no HR, OR, IRR, or SIR/SHR provided (Pan et al., 2019); study investigated relationships between irregular diet and cancer, but not EC specifically; and (Xie et al., 2020) the study investigated relationships between dietary habits and cancer, but did not include irregular diet.

### Quality evaluation

The Newcastle–Ottawa scale (NOS) was used to evaluate the quality of the studies included (Wells et al., 2020). The scale evaluates three aspects of studies: selection of participants and measurement of exposure, comparability, and assessment of outcomes and adequacy of follow-up. In the current analysis low-quality studies were those with scores of 0–3, moderate-quality studies were those with scores 4–6, and high-quality studies were those with scores of 7–9.

### Data extraction

A standardized data extraction sheet was developed in Excel (Microsoft Corporation). Two investigators (Jiayun Guan and Xixin Pan) independently extracted the first author, year, location, study design, sample size, sex, age, rage of BMI, type of Esophageal cancer, causes of irregular diet, sample size, RR, and 95% confidence intervals in the fully adjusted model from the studies. Another investigator (Shenghang Ruan) checked the extracted data. Any discrepancies were discussed with Xiaoxiang Rong and the final decision is made by Xiaoxiang Rong.

### Statistical analysis

Meta-analyses were performed by calculating RRs with 95% CIs for EC incidence using a random-effects model. Heterogeneity across studies was assessed using the Q statistic with its *p* value and I^2^ statistic (Higgins et al., 2003). The I^2^ statistic is used to quantify the proportion of total variation in the effect estimation that is due to between-study variations. An I^2^ > 50% indicates significant heterogeneity (Higgins and Thompson, 2002). A two-sided *p* value <0.05 was considered statistically significant. A funnel plot was constructed to assess the risk of publication bias, and the robustness of the pooled RR for the occurrence of EC with irregular diet was validated by sensitivity analysis. Subgroup analyses of populations with different characteristics were also performed to investigate relationships between irregular diet and risk of EC. All analyses were performed using Stata statistical software versions 14.0 and 16.0.

## Results

### Study selection and characterization

**FIGURE 1:**
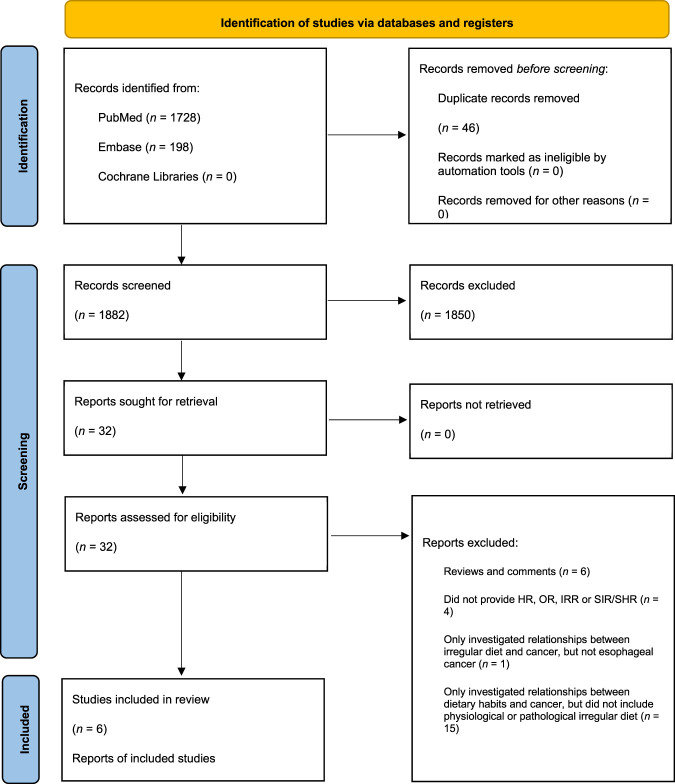
Study selection process.

A total of 1928 articles were retrieved from PubMed, Cochrane Libraries, and Embase. 1882 remained after the removal of deduplicates. After reading the titles and abstracts of the articles, 1850 that did not meet the requirements were initially excluded. The full texts of the remaining 32 articles were read. Among those 32 articles which included other diet issues, 26 were excluded because they were reviews or comments (Manoogian et al., 2019), did not provide HR, OR, IRR, or SIR/SHR (Xie et al., 2020), only investigated relationships between irregular diet and cancer, but not EC (Sung et al., 2021), or only investigated relationships between dietary habits and cancer, but did not include irregular diet (Mellemkjaer et al., 2015). Specific screening procedures and reasons for exclusion are shown in Figure 1.

Six relevant articles (Mellemkjaer et al., 2001; Sun et al., 2010; Brewster et al., 2015; Mellemkjaer et al., 2015; Yeh et al., 2021; Zhang et al., 2021), which were published between 2001 and 2021, are shown in Table 1. Among the involved studies, two were performed in China, one study was conducted in Sweden, Denmark and Finland, the other 3 studies were respectively performed in the United States, Scotland, and Denmark. Among the 6 papers, five were cohort studies and one was a case-control study, with a total of 82,296 participants. The causes of irregular diet were not all the same across the six studies, with two anorexia nervosas, two eating disorders, one irregular eating, and one unrestrained eating behavior. The estimated quality of all included studies was high. All included studies obtained an NOS of at least 6 points (Table 2).

**TABLE 1:** Basic characteristics of the studies included in the meta-analysis.

Author	Year	Location	Study Design	Sample size	Sex (Male/Female)	Age	Rage of BMI	Type of Esophageal cancer (ESCC/EAC)	Causes of irregular Diet	RR (95%CI)
Mellemkjaer, L.	2015	Sweden, Denmark, Finland	Cohort study	24,332	1678/22654	ND	ND	3/2	Anorexia nervosa	5.10 (1.80–14.60)
Zhang, Y.	2021	America	Cohort study	44,382	0/44382	30–55	21–34.36	26/52	Unrestrained eating behavior	1.62 (1.01–2.62)
Brewster, D. H.	2015	Scotland	Cohort study	3617	350/3267	ND	ND	7/0	Eating disorder	6.12 (2.46–12.60)
Yeh, H. W.	2021	Taiwan, China	Cohort study	6628	1496/5132	≥20	ND	ND	Eating disorder	5.32 (1.07–26.49)
Xibin Sun	2010	China	Case-control	1000	280/720	ND	ND	ND	Irregular meal times	4.80 (2.56–8.99)
Mellemkjaer, L.	2001	Denmark	Cohort study	2337	186/2151	ND	ND	ND	Anorexia nervosa	20.00 (0.50–111.00)

ND, not described.

**TABLE 2:** Quality assessment of the studies included in the meta-analysis.

Study	Selection	Comparability control for important factor	Exposure	Total
Xibin Sun 2010	**	**	**	6
Zhang, Y. 2021	***	**	***	8
Brewster, D. H. 2015	**	**	**	6
Yeh, H. W. 2021	***	**	***	8
Mellemkjaer, L. 2015	****	*	**	7
Mellemkjaer, L.2001	**	*	***	6

### Data evaluation and quality control

**FIGURE 2:**
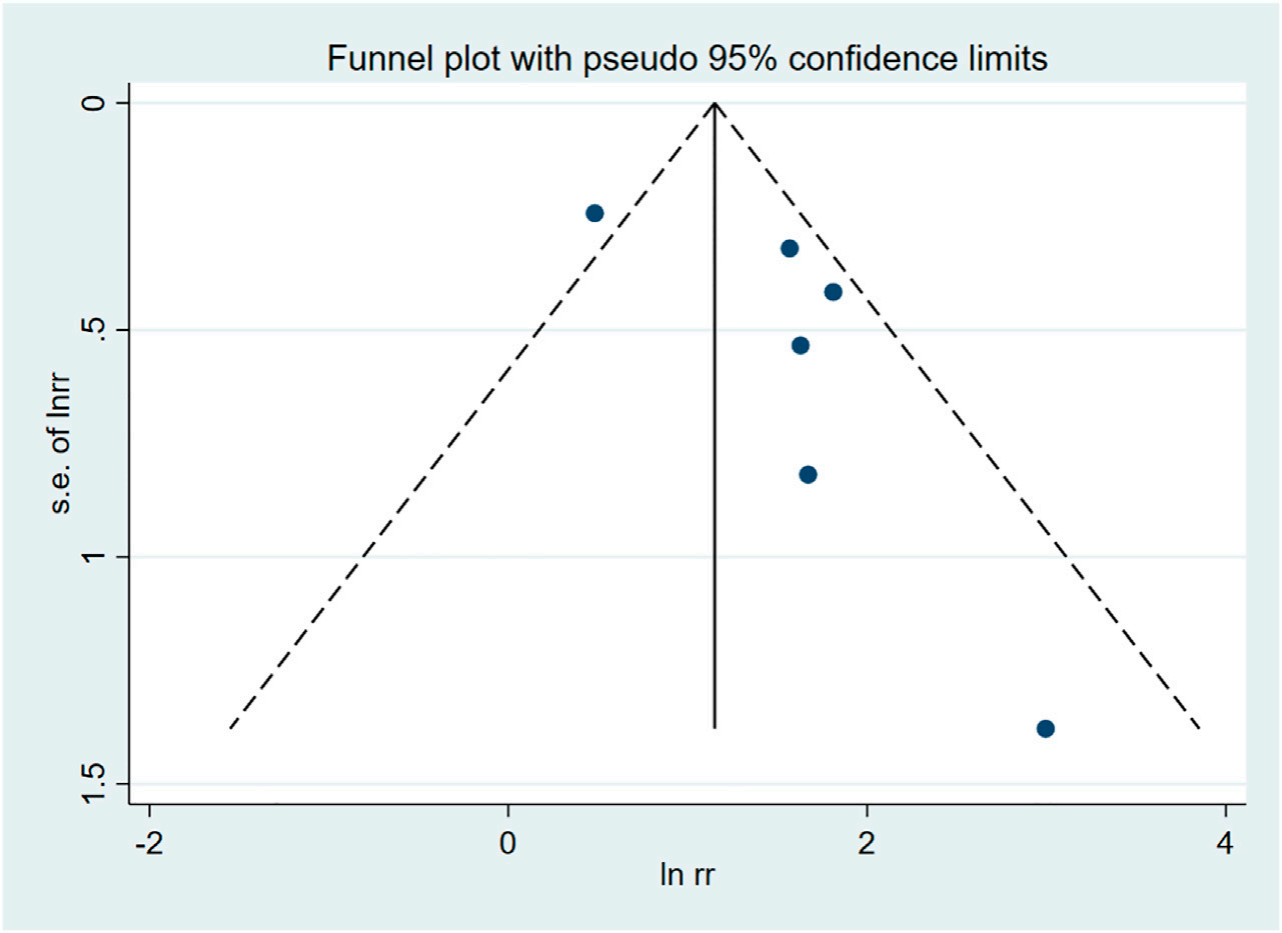
Funnel plot of publication bias.

**FIGURE 3:**
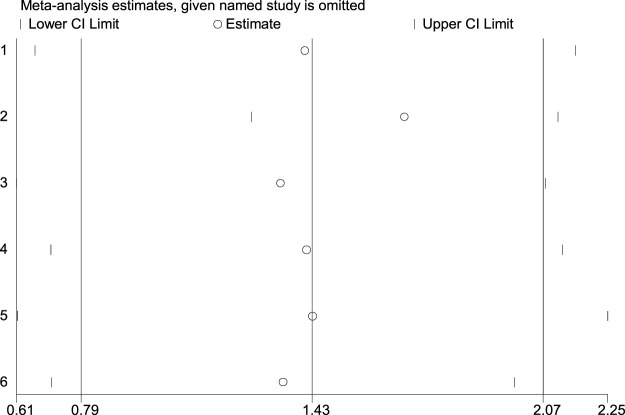
Sensitivity analysis of the association between irregular diet and risk of esophageal cancer.

Funnel plot was used to evaluate the publication bias (Figure 2). Egger’s regression test was conducted to analyze the symmetry of the funnel plot. None of the articles demonstrated publication bias (*p* = 0.116 (Figure 3)). And in sensitivity analysis, we found that the integrated RR did not change significantly, revealing the robustness of the pooled results.

### Results of Meta-Analysis

**FIGURE 4:**
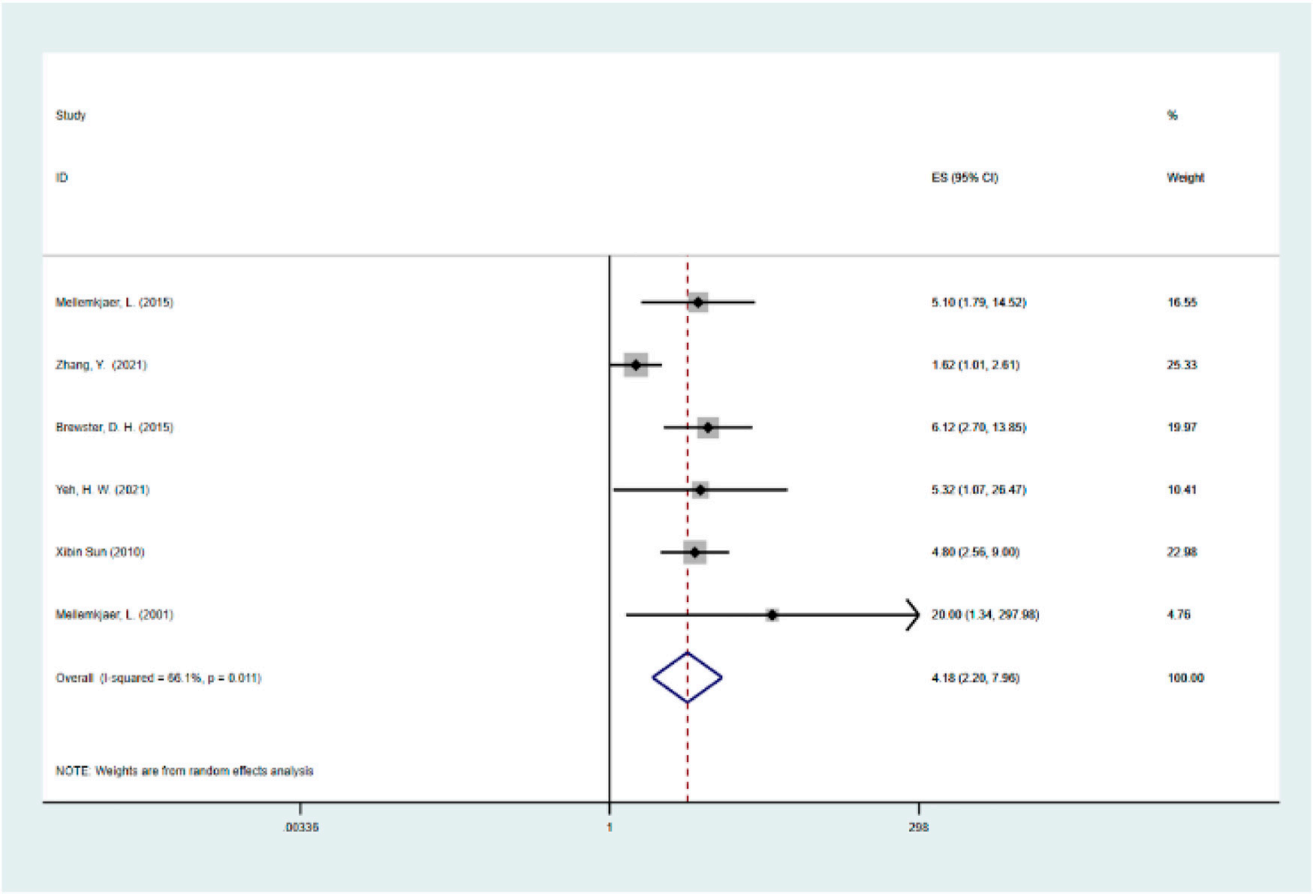
Forest plot of irregular diet and the incidence of esophageal cancer.

The summary RR was 4.18 (95% CI, 2.19–7.96; I2 = 66.1%; *p* = 0.011), with moderate heterogeneity (Figure 4). This result means that the risk of esophageal cancer is 4.18 times higher for those who with irregular diet than those who with regular diet. Our analysis showed that the new combined RR was similar with the original RR and irregular diet may lead to increased risk of esophageal cancer. And our results are consistent with the conclusions of the included literature studies.

Subgroup analyses because of severe heterogeneity, further analyses were performed based on subgroup analyses to find out potential sources of heterogeneity. Results of subgroup analyses are shown in Table 3. When the study was classified according to the nature of irregular diet, the results showed that the relative risk of esophageal cancer in the Disease-causing group increased by 5.99 times, while the Non-disease-causing group only increased by 2.73 times. When the included studies were divided into Asian and non-Asian subgroups based on geographical distribution, the RR increased by 4.10 times in the Asian group and 4.87 times in the non-Asian group. In the research method subgroup, the Case-control group had a 1.57-fold improvement in RR compred to a 4.19-fold improvement in the Cohort study group. This result proved that irregular diet caused by disease is more likely to lead to the occurrence of esophageal cancer. We also performed subgroup analyses by geographical distribution of studies, research method, and Occupation of the study population.

**TABLE 3:** Subgroup analysis of irregular diet and esophageal cancer.

Items	Number of cohorts	RR	95% CI	I^2^	*p*-value
The nature of irregular diet					
Non-disease-causing	2	2.73	0.94–7.92	86.3%	0.07
Disease-causing	4	5.99	3.35–10.75	0.0%	0.83
Geographical distribution of studies					
Asian	4	4.10	1.59–10.61	74.5%	0.008
Non-Asian	2	4.87	2.71–8.73	0.0%	0.91
Research method					
Case-control	1	1.57	0.94–2.19	—	—
Cohort study	5	4.19	1.84–9.53	67.9%	0.01
Occupation of the study population					
Nurse	1	0.48	0.006–0.96	—	—
Non-nurse	5	5.41	3.53–8.29	0.0%	0.89

## Discussion

### The main findings and possible mechanisms responsible for the findings of this study

This study reported the first meta-analysis of associations between irregular diet and risk of esophageal cancer to our knowledge. Our study found that irregular diet increases the risk of esophageal cancer, whether or not the irregular diet is caused by the disease. This finding is consistent with previous studies (Brewster et al., 2015; Zhang et al., 2021).

The mechanisms by which irregular diet increases the risk of EC are not well understood. Moreover, the associations we observed are likely explained by more than one mechanism. Previous studies have shown that irregular diet is an important components regulating the circadian clock, and irregular diet can cause circadian misalignment which can cause substantial plasma proteome changes, especially for proteins associated with pathways involved in cancer (Depner et al., 2018; Srour et al., 2018) In addition, people with irregular diet can have behaviors such as indulging late-night eating, eating large meals and unrestrained eating behavior, which can lead to gastroesophageal reflux disease and subsequent Barrett’s esophagus, the most common esophageal precancer (McCullough and Willett, 2006). At the same time, those with eating large meals or unrestrained eating behavior can trigger molecular mechanisms giving rise to leptin due to irregular diet (Vasselli et al., 2013; Chang et al., 2020; Mendoza-Herrera et al., 2021). And an increased level of leptin was associated with an increased risk for esophageal cancer. Moreover, vomiting is known to be a common symptom behavior of EDs, which might result in damage to the esophageal mucosa, thereby increasing the occurrence of esophageal cancer (Graham and Yamaoka, 1998; Reba et al., 2005).

### Subgroup analysis discussion

Data from 6 studies in our meta-analysis indicated that irregular diet increases the risk of EC. However, there was moderate heterogeneity of the summary RR of our meta-analysis, and we performed a subgroup analysis for this. In the subgroup analysis, we found significant heterogeneity in the Non-disease-causing group from the subgroup analysis, which may be due to the different behavior of irregular diet in the two studies in this group, one with unrestrained eating behavior and the another with irregular meal time. The heterogeneity of these two behaviors may be due to the different mechanisms of the two behaviors leading to the increased risk of esophageal cancer. The former is more inclined to be related to metabolic switching and cellular stress resistance (de Cabo and Mattson, 2019), while the latter is more inclined to increase the burden on the digestive tract. Another important source of heterogeneity was occupation, with the nurse group being the main source of heterogeneity. Because the research population of this study is all female nurses in the United States. Nurses differ from the masses in that they need to be engaged in shift-work which make them more prone to cancer (Straif et al., 2007). And within subgroups by geographic location and study methodology, heterogeneity may arise from differences in the study population, study design, and methodology. The above three factors that produce heterogeneity may be the source of heterogeneity in the results of this study. However, evidence of heterogeneity still existed even after subgroup and sensitivity analyses, which suggested that our meta-analysis results were slightly influenced by heterogeneity. Considering the existence of heterogeneity, our results need to be further confirmed by large-scale clinical trials.

### Implications for clinical practice

In the current meta-analysis irregular diet is associated with an increased risk of EC. This has important clinical significance and can guide clinicians treating EDs or anorexia nervosa; specifically, to ensure their regular diet to the greatest extent possible, and reduce their risk of EC. ED-associated behaviors are relatively common, and EC has high morbidity and mortality. Thus, the focus should be on prevention. As the first step of prevention, it should be recognized that irregular diet is a risk factor for EC. Correcting the irregular diet of patients and reminding people to eat regularly may reduce the incidence of EC.

### Strengths and limitations of the meta-analysis

The present meta-analysis had several strengths. To the best of our knowledge it is the first to investigate associations between irregular diet and the risk of EC. The meta-analysis can provide a valuable reference for the clinical prevention and treatment of EC.

However, the meta-analysis also had some limitations. First of all, it is known that, EC is mainly divided into Esophageal squamous cell carcinoma (ESCC) and Esophageal adenocarcinoma (EAC). ESCC is the mainly pathology type in Asian while EAC is the mainly pathology type in western countries (Hongo et al., 2009). It is important to further explore the association between irregular diet and ESCC and EAC. But there were insufficient data to perform subgroup analyses based on pathological type of EC. Second, the aggregated data of our study were originated from observational studies, thus, the association between irregular diet and the risk of EC remains speculative. Meanwhile, since only cohort studies and case–control studies were selected, a large number of studies had to be excluded either during the screening of titles and abstracts or during the assessment of full-texts. What’s more, we focused on the quite specific diet issue without information of other diet issues. In that case, when we combine all the database together, it is not sure whether the negative correlation datapoint in one database would be the positive correlation datapoint in the other database. As a result, the limitation of source information will lead to biased analysis. Furthermore, all the studies included were also all conducted in northern Europe or China, with the exception of one conducted in the United States, so the results have geographical limitations and cannot be readily generalized to the entire world. If additional relevant prospective studies are conducted in the future, relationships between physiological or pathological dietary irregularities and the occurrence of EC can be more accurately explained.

## Conclusion

The present meta-analysis indicates that a irregular diet increases the risk of EC. The analysis had geographical limitations, and more research is needed to facilitate broader generalization of our conclusion.

## Data Availability

The raw data supporting the conclusion of this article will be made available by the authors, without undue reservation.
