# Supplementary material for: Relationship between irregular diet and risk of esophageal cancer: A meta-analysis
Source: Front Genet. 2022 Oct 5;13:1004665. doi: 10.3389/fgene.2022.1004665 (PMC9581389; doi:10.3389/fgene.2022.1004665)
Supplement: Supplementary file 1 [file Table1.docx]

**Supplementary table 1.** Detailed search strategy in PubMed database.

| Search number | Query | Results |
| --- | --- | --- |
| 4 | ((#1) AND (#2) AND (#3)) | 1,775 |
| 3 | ((risk[Title/Abstract]) OR (risk[MeSH:noexp]) OR (mortality[Title/Abstract]) OR (mortality[MeSH:noexp]) OR (cohort[Title/Abstract])) | 3,485,616 |
| 2 | ((Esophageal Cancer [MeSH Major Topic]) OR (Esophageal Neoplasm[Title/Abstract]) OR (Cancer of Esophagus[Title/Abstract]) OR (Cancer of the Esophagus[Title/Abstract]) OR (Esophagus Cancer[Title/Abstract]) OR (Esophagus Cancers[Title/Abstract]) OR (Esophageal Neoplasms [Title/Abstract]) OR (Esophageal Cancers[Title/Abstract]) OR (Cancers[Title/Abstract]) OR (Cancer[Title/Abstract])) | 2,089,959 |
| 1 | ((eating disorders [MeSH Major Topic]) OR (Anorexia nervosa[Title/Abstract]) OR (Anorexia Nervosas[Title/Abstract]) OR (eating [Title/Abstract]) OR (Eating Disorder[Title/Abstract])) | 101,485 |
